# Functional and Structural Analysis of Maize Hsp101 IRES

**DOI:** 10.1371/journal.pone.0107459

**Published:** 2014-09-15

**Authors:** Augusto Samuel Jiménez-González, Noemí Fernández, Encarnación Martínez-Salas, Estela Sánchez de Jiménez

**Affiliations:** 1 Departamento de Bioquímica, Facultad de Química, Universidad Nacional Autónoma de México, México DF, México; 2 Centro de Biología Molecular Severo Ochoa, Consejo Superior de Investigaciones Cientificas –Universidad Autónoma de Madrid, Madrid, Spain; The John Curtin School of Medical Research, Australia

## Abstract

Maize heat shock protein of 101 KDa (HSP101) is essential for thermotolerance induction in this plant. The mRNA encoding this protein harbors an IRES element in the 5′UTR that mediates cap-independent translation initiation. In the current work it is demonstrated that hsp101 IRES comprises the entire 5′UTR sequence (150 nts), since deletion of 17 nucleotides from the 5′ end decreased translation efficiency by 87% compared to the control sequence. RNA structure analysis of maize hsp101 IRES revealed the presence of three stem-loops toward its 5′ end, whereas the remainder sequence contains a great proportion of unpaired nucleotides. Furthermore, HSP90 protein was identified by mass spectrometry as the protein preferentially associated with the maize hsp101 IRES. In addition, it has been found that eIFiso4G rather than eIF4G initiation factor mediates translation of the maize hsp101 mRNA.

## Introduction

Maize heat shock protein of 101 kDa (HSP101) mRNA encodes a chaperone essential for thermo-tolerance induction in maize (*Zea mays L.*) [1]. We have previously shown that this mRNA harbors an Internal Ribosome Entry Site (IRES) element on its 5′ untranslated region (5′UTR) which mediates cap-independent translation initiation in cell free lysates [2]. In the later study, cap-dependent translation was inhibited through the proteolysis of eIF4G induced by the action of the Lb protease.

Most eukaryotic mRNAs contain a cap structure (m^7^GpppN, where N represents any nucleotide) at its 5′UTR end. The cap structure is recognized by the eIF4F initiation factor consisting of eIF4A, eIF4G and eIF4E [3]. The eIF4E protein binds directly to the cap structure of the mRNA. Therefore, it is essential for cap-dependent translation initiation and recruitment of the translational machinery.

IRES elements are nucleotide sequences, often located within the 5′ UTR of some mRNAs, which mediate initiation of translation under conditions that compromise cap-dependent initiation [4], [5]. However, IRES elements do not present extensive sequence similarities, although some IRES contain short sequences acting as conserved motifs that are exposed in the IRES structure guiding its recognition by the translational machinery [6], [7].

Under certain stress conditions including heat shock, cap-dependent translation initiation is severely inhibited [8]. On the other hand, cellular mRNAs containing IRES are activated by physiological stimulus under stress conditions that generally compromise cap-dependent translation, such as gamma-radiation, apoptosis, heat stress or viral infections [9], [10]. Coincident with these stress signals, translation of a subset of mRNAs encoding proteins such as transcription factors, growth factors and survival proteins that are functional under cellular stress [5], [11] remain unabated, providing selective advantage to mRNAs that initiate translation using a cap-independent mechanisms [12].

IRES-mediated translation depends on its primary sequence as well as on its RNA structural organization. Generally, deletions, insertions or substitutions can severely decrease its activity [13], although a few examples have been reported in which mutations increase its translation efficiency [14], [15]. Therefore, IRES elements provide an alternative mechanism for translation initiation that depends on the recognition of the RNA by the 40S ribosomal subunit to form the active translational complex [16]. This process is usually aided by translation initiation factors (eIFs) and IRES-transacting factors (ITAFs), with the exception of the IRES element present in the intergenic region of dicistroviruses that is independent of eIFs [17].

Sessile organisms, such as plants, cannot avoid adverse environmental conditions. Therefore, diverse mechanisms for gene expression regulation that allow adequate responses to stress conditions have been developed in order to facilitate translation of certain factors mediating survival in response to adverse environments [18]. IRES-dependent translation mechanisms have been identified in viral RNAs that infect plants [19]–[24] as well as in cellular mRNAs encoding the heat shock protein HSP101 or the alcohol dehydrogenase (ADH) [2], [25]. However, little is known about the RNA structural organization of these IRES elements.

To gain insights about the relationship between RNA structure and IRES function of plant mRNAs, we carried out a functional and structural analysis of maize hsp101 IRES. These studies identified critical stem-loops needed for IRES activity. In addition, mass spectrometry identification of factors bound to hsp101 IRES element revealed HSP90 as a factor specifically associated to the IRES competent complex under cap-independent conditions. Finally, *in vitro* translation of hsp101 mRNA showed selectivity for eIFiso4G rather than eIF4G translation initiation factor.

## Results

### Functional analysis of the maize hsp101 IRES

**Figure 1:**
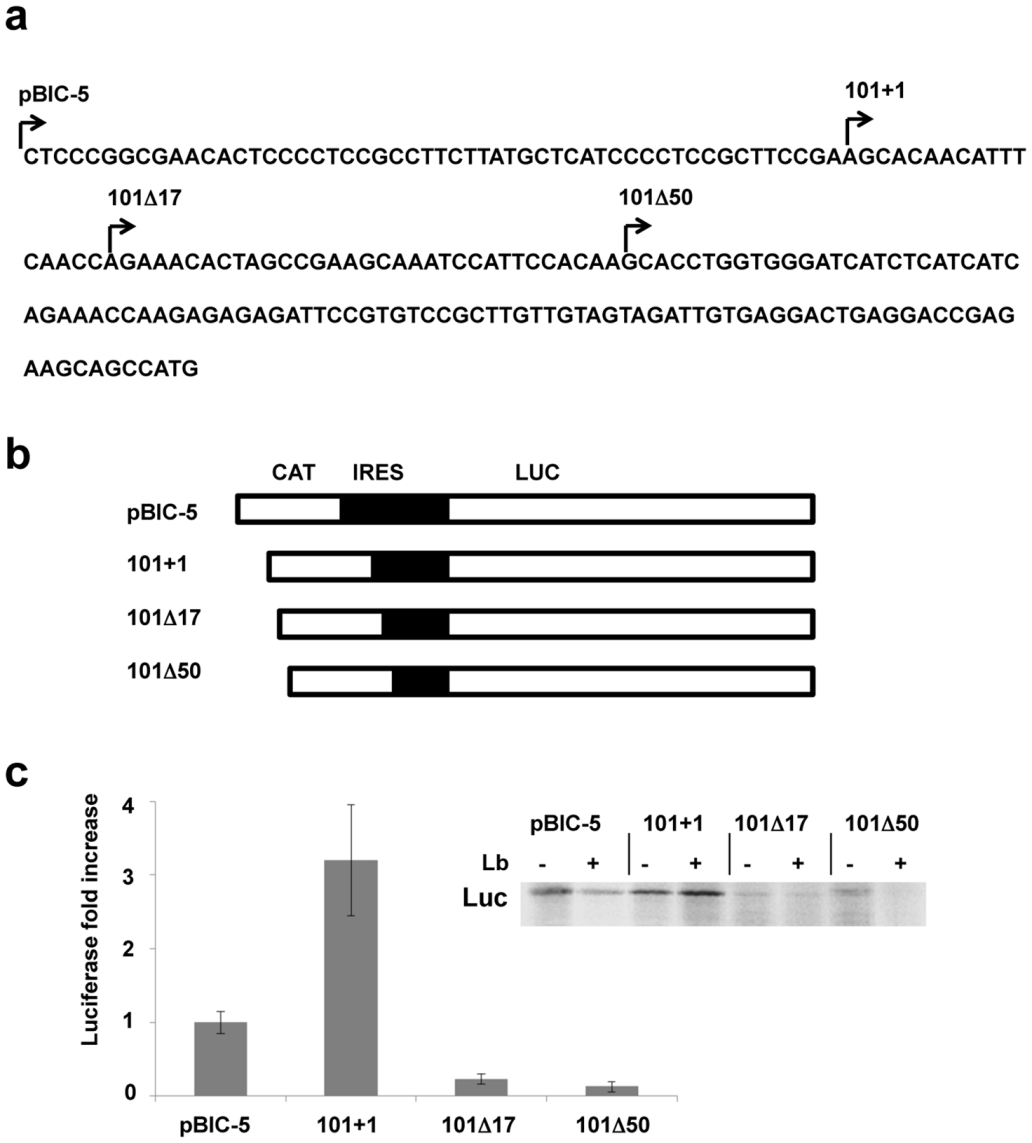
Functional analysis of the maize hsp101 IRES. a) Maize *Hsp101* 5′UTR sequence. Arrows indicate the first nucleotide of the fragments used to identify the functional IRES region. b) Schematic representation of the bicistronic constructs pBIC-5 plasmid was used as positive control. *Hsp101* fragments (sense orientation) were inserted between the chloramphenicol acetyl transferase (CAT) and luciferase (Luc) reporter genes. c) Translation efficiency of the mutant IRES elements in RRL. Equal amounts of *in vitro* synthesized RNAs pBIC-5, 101+1, 101Δ17 or 101Δ50 were used to program translation using Lb-untreated (-) RRL or Lb-treated (+) RRL. Proteins were resolved by SDS–PAGE and visualized by autoradiography of dry gels. Synthesis of luciferase driven by the indicated IRES elements in the presence or absence of the Lb protease is shown on the insert. The histogram shows the intensity of luciferase translation efficiency driven by the indicated RNAs in the presence of Lb (cap-independent).

In order to identify the minimal region of the maize hsp101 IRES driving internal initiation of translation, 5′end deletions of the IRES sequence inserted in pBIC-5 construct were generated. The pBIC-5 construct, containing an active maize hsp101 IRES [2], harbors 60 nts belonging to the genomic region upstream of the transcription start site (+1) (Figure 1a). Thus, to verify whether these extra 60 nts altered the hsp101 IRES activity, a construct named 101+1 (starting at the +1 transcription start site) was generated. In addition, 5′end deletions of 77 and 110 nts were introduced in constructs 101Δ17 and 101Δ50, respectively (Figure 1a). These sequences were inserted in the pBIC bicistronic vector in which translation of the first reporter gene (CAT) is cap-dependent while translation of the second reporter gene (LUC) is cap-independent (Figure 1b). pBIC-5, 101+1, 101Δ17 and 101Δ50 RNAs synthesized *in vitro* were translated using the rabbit reticulocyte lysate (RRL) translation system in a cap-dependent manner. In parallel, translation of these RNAs was analyzed in lysates expressing the Lb protease of FMDV, as previously done with the pBIC-5 IRES sequence [2]. This protease cleaves eIF4G [26], rendering the cell-free translation system cap-independent. In the presence of Lb protease, LUC protein was synthesized from RNAs pBIC-5 and, particularly, 101+1 (insert in Figure 1c). In contrast, very low levels of luciferase were detected in lanes loaded with reaction products driven by 101Δ17 RNA. The fold-increase of LUC protein synthesis was plotted to compare translation initiation efficiency among the different IRES constructs. To this end, the intensity of LUC polypeptide was normalized to the value obtained for pBIC-5 RNA (the construct with proven IRES activity) set to 100% (Figure 1c). Translation efficiency of luciferase from mRNA 101+1 was three fold higher than the control RNA pBIC-5 in the presence of Lb. In contrast, translation efficiency of mRNAs 101Δ17 and 101Δ50 decreased severely (83% and 90%, respectively) in comparison to the control pBIC-5 (Figure 1c). These results demonstrated that the complete 5′UTR sequence of hsp101 RNA was required for IRES-dependent translation initiation under conditions that compromise cap-dependent initiation, here illustrated by Lb protease coexpression.

Given that the sequence in 101+1 RNA was identified as the authentic maize hsp101 IRES functional region, we analyzed the secondary structure of this RNA in order to identify potential RNA motifs mediating internal initiation, likely shared with other IRES elements.

### Secondary structure of maize IRES hsp101

**Figure 2:**
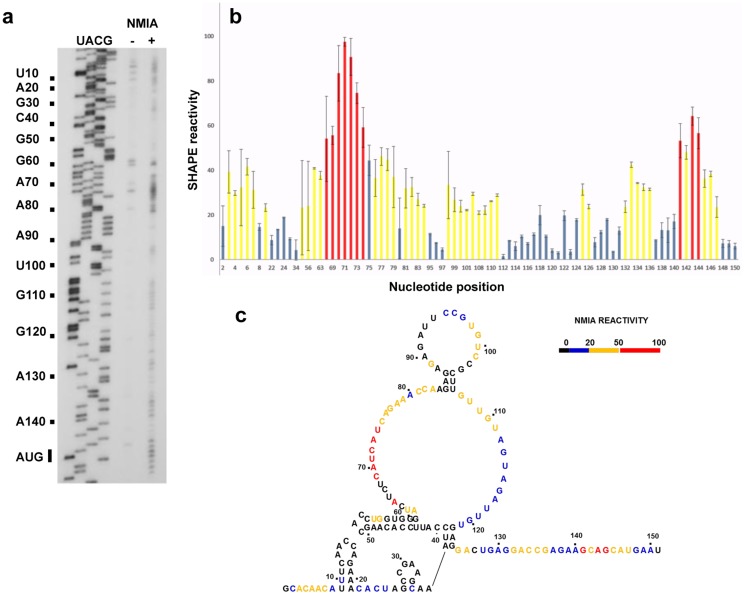
SHAPE structural analysis of maize hsp101 IRES. a) Primer extension analysis of 101+1 RNA. The first four lanes (UACG) show hsp101+1 sequence, obtained with the 5′end labeled primer. Lanes 5 and 6 show the primer extension products obtained with 101+1 RNA untreated (−) or treated (+) with NMIA, respectively b) SHAPE reactivity of 101+1 RNA. Reactivity was normalized relative to the full-length product (set to 100%). Blue bars represent values <20; yellow bars represent values <50 and red bars represent values >50. c) Maize hsp101 IRES secondary structure. Colors of the nucleotides reflect SHAPE reactivity values.

To characterize the secondary structure in solution of maize hsp101 functional IRES, 101+1 RNA was treated with N-methylisatoic anhydride (NMIA), which reacts with the single-stranded 2′hydroxylic groups of nucleotides to form a stable 2′-O-aduct [27]. This modification was identified by primer extension reaction as reverse transcriptase (RT) stop sites [28] (Figure 2a). The intensity of RT stops were quantified by densitometry and then normalized to the intensity of the full-length product obtained in each RT reaction set to 100%. Four ranges of reactivity were chosen, colored black, blue, yellow and red (0, >20, >50 and <50, respectively) in Figure 2b. Secondary structure of the functional maize hsp101 IRES was obtained by using the values for NMIA reactivity in the mFOLD software; nucleotides are presented in a color scale reflecting their reactivity to NMIA (Figure 2c). The RNA structure revealed a large proportion of nucleotides that are reactive to NMIA, thereby unpaired, with the exception of 3 stem-loops located at the 5′ end of the RNA. The first hairpin comprises nucleotides 9–21, thus including most of the 17 nucleotides whose deletion leads to a loss of IRES activity. Together, these results demonstrate the importance of this hairpin for internal translation initiation. Furthermore, consistent with the defective phenotype of the deletion of the first hairpin, a construct lacking 50 nucleotides at the 5′end eliminating the 3 hairpins also yielded fully defective IRES elements.

### Isolation of RRL proteins bound to IRES under cap-independent conditions

**Figure 3:**
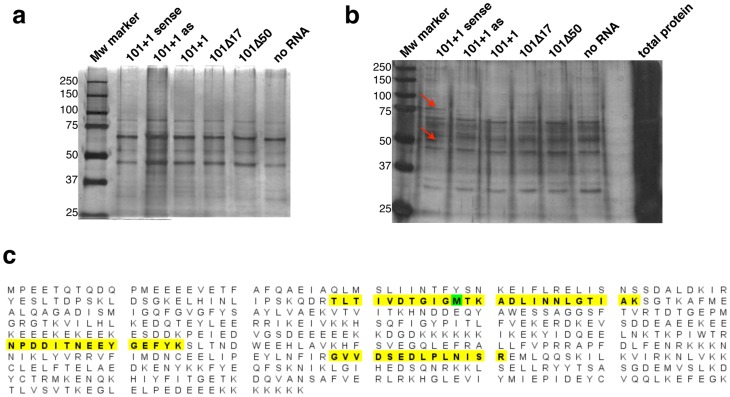
Identification of proteins associated to maize hsp101 IRES. Purification of IRES-protein complexes using RRL translation system (a), or Lb-treated RRL translation system (b) with RNAs indicated at the top. Equal amounts of samples containing the proteins that coprecipitated with the indicated RNAs were loaded on SDS-PAGE and detected by silver staining. Red arrows depict two bands specifically copurifying with 101+1 sense RNA using the Lb-treated RRL. c) Sequence of the 75 kDa protein that binds to maize hsp101 IRES. The 75 KDa band (indicated by an arrow in lane 2 of panel b) was sequenced and identified as the molecular chaperone HSP90. The results yielded 4 unique peptides (shaded in yellow), 4 unique spectra, 5 total spectra, 54/565 amino acids (10% coverage).

Once the secondary structure of the functional region of maize hsp101 IRES was elucidated, we set up to identify candidate proteins potentially interacting with this element. For this, poly(A)-tailed RNAs were generated to capture the proteins that bind to maize hsp101 IRES taking advantage of the poly(A) affinity to oligo dT cellulose. In addition, untreated and Lb protease-treated lysates were used to favor cap-dependent or cap-independent conditions, respectively. No differences were observed in the pattern of proteins purified with the RNAs tested using untreated lysates (Figure 3a). In contrast, two bands of ∼50 kDa and ∼75 kDa were specifically associated to 101+1 sense RNA using the Lb-treated RRL (red arrows in Figure 3b). Notably, these bands were absent in samples pulled down with the antisense 101+1as RNA. These results indicate that the 75 and 50 kDa factors specifically interact with the authentic hsp101+1 RNA. Lack of binding of these factors to the RNAs derived from pGEM-T 101+1, 101Δ17 and 101Δ50 could be explained at least in part by differences in the RNA structure induced by 30 nt polylinker sequence located at their 3′end that preclude their interaction. Mass spectrometry (LS/MS/MS) identification of these bands revealed that the ∼75 kDa band was the molecular chaperone HSP90 (Figure 3c), as demonstrated by the presence of 4 unique peptides (yellow marks in Figure 3c). The second protein could not be unambiguously identified. We conclude that HSP90 protein forms part of the maize hsp101 IRES translational complex assembled under cap-independent conditions.

### Functional relevance of HSP90, eIF4G and eIFiso4G in maize hsp101 translation

**Figure 4:**
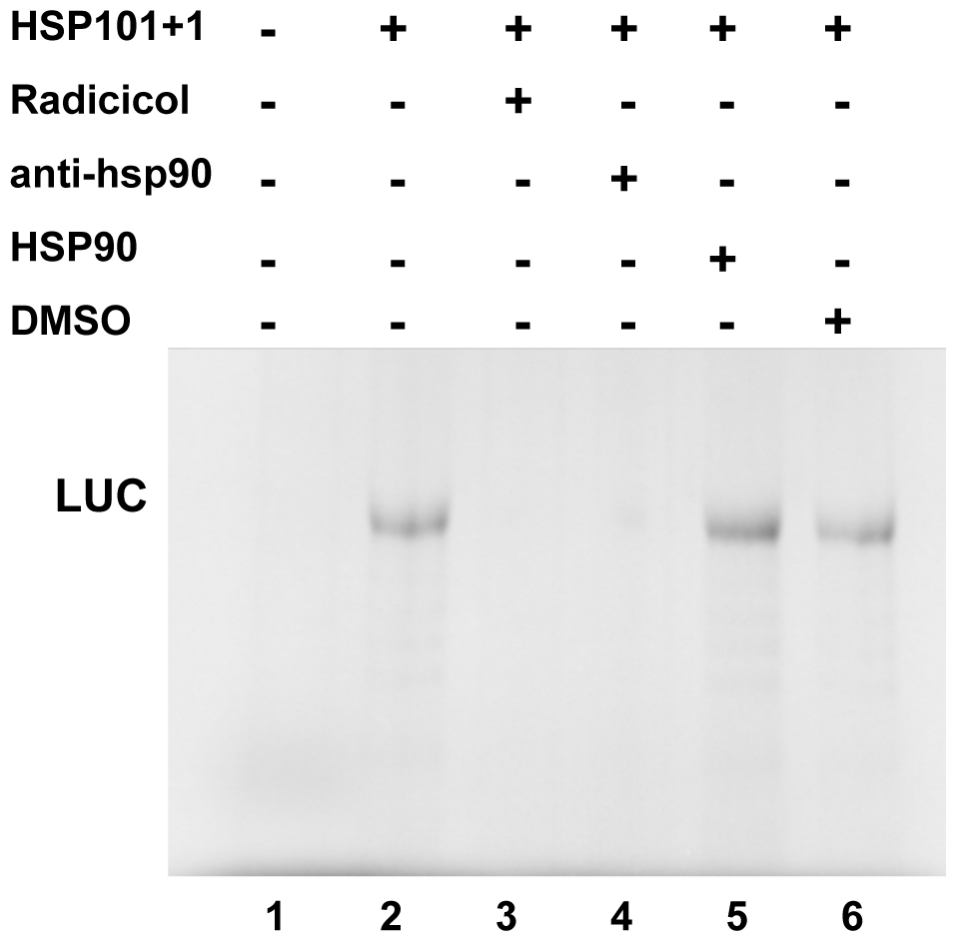
Effect of HSP90 protein on hsp101+1 translation initiation. Equal amounts of *in vitro* synthesized RNAs 101+1 were used to program translation using RRL in the presence of Radicicol (lane 3), anti-HSP90 (lane 4), HSP90 protein (lane 5), or DMSO (lane 6). Proteins were resolved by SDS–PAGE and visualized by autoradiography of the dry gel. Synthesis of luciferase driven by the indicated IRES elements in the presence of the Lb protease (cap-independent).

To confirm that the HSP90 protein was involved in maize hsp101 IRES-driven translation, we conducted cap-independent *in vitro* translation assays. Additionally, two potential inhibitory reagents were added to the translation system. One of them, radicicol is an HSP90 specific ligand [29]. The second one was a specific antibody HSP90 (anti-HSP90 Ab). Under cap-independent conditions both, Radicicol and anti-HSP90 Ab inhibited translation of the 101+1 mRNA was (Figure 4). These data revealed the importance of HSP90 for maize hsp101 mRNA translation. However, addition of commercial purified human HSP90 protein to the system did not affect translation efficiency of 101+1 (Figure 4), suggesting that the level of HSP90 protein present in the lysate is sufficient for IRES activity.

**Figure 5:**
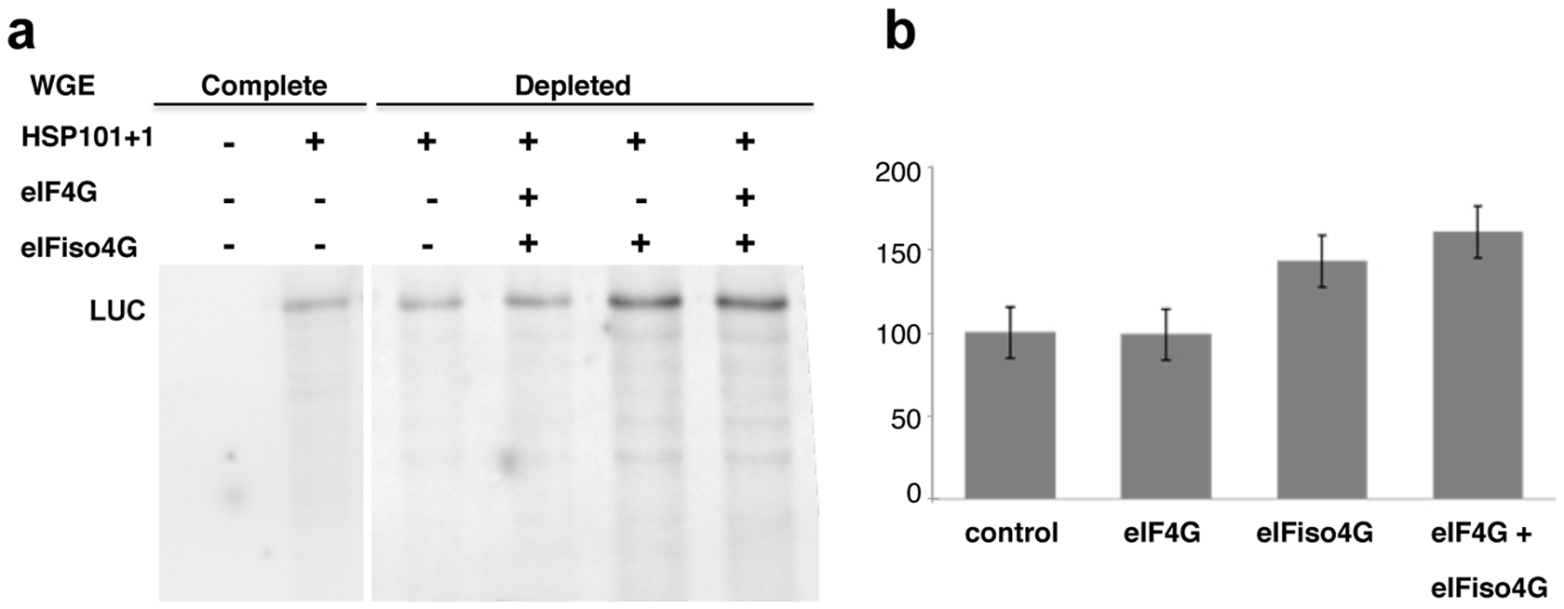
Hsp101+1 translation in a wheat system (WGE) depleted from eIF4E, eIFiso4E, eIF4G and eIFiso4G. a) Maize hsp101 translation in the complete WGE system. Lane 1: background signal obtained without added RNA (negative control); lane 2: hsp101+1-driven translation products (positive control). Maize hsp101 translation in the depleted WGE system: hsp101+1-driven translation (3); hsp101+1 translation adding recombinant eIF4G factor (4); hsp101+1 translation adding recombinant eIFiso4G factor (5); hsp101+1 translation adding both eIF4G and eIFiso4G (6). b) Histogram of cap-independent translation efficiency of RNA 101+1 when eIF4G and eIFiso4G are added to the system. Values represent the mean (±SD) of three independent assays.

Plants have two isoforms of eIF4G, being eIFiso4G preferentially used for translation initiation of mRNAs with 5′UTR stable secondary structure, with hypermethylated cap structures or actively involved in IRES-dependent translation [30], [31]. Thus, since many viral and cellular IRES elements depend on eIF4G factor, we investigated the role of eIF4G and eIFiso4G factors for maize hsp101 translation. For this purpose, *in vitro* translation assays were performed using the wheat germ system (WGE) depleted from either eIF4F or eIFiso4F (eIF4E-eIF4G and eIFiso4E-eIFiso4G, respectively) using m^7^GTP-sepharose. Then, addition of wheat recombinant eIF4G or eIFiso4G to the translation system was conducted to determine whether any of these factors affected hsp101 IRES-directed translation. No significant difference in hsp101 translation efficiency was observed when eIF4G was added to the system compared to the depleted lysate. However, translational efficiency increased by 40% when eIFiso4G was added to the translation system compared to the control. Furthermore, hsp101 translation efficiency increased by 60% when both factors were added to the system (Figure 5). These results suggest that there is selectivity on the use of factors used to initiate translation, and that maize hsp101 IRES has preference for eIFiso4G over eIF4G to form the active translational complex.

## Discussion

Eukaryotic mRNA translation initiation depends on the cap structure located at the 5′end [3]. However, under conditions that inhibit cap-dependent translation, activation of IRES-dependent translation facilitates cell survival. IRES elements, usually located at the 5′ UTR of some mRNAs of eukaryotic cells and their viruses, do not present extensive similarities in their primary sequence or RNA structure. Therefore, definition of the boundaries of IRES elements as well as their structure has to be experimentally delimited case by case. This can be accomplished by functional analysis of the candidate sequences, using cap-independent translation conditions [11]. Earlier studies have shown that some IRES elements are active in heterologous systems [20], [32]–[34]. In agreement with this, the maize hsp101 mRNA contains an IRES element which is active under eIF4G cleavage induced by the Lb protease activity in RRL [2]. In the current study, and using the same system, it is shown that deletions or insertions at the 5′end of the authentic hsp101 5′UTR affect the translation efficiency. Thus, the entire 5′UTR sequence of the heat shock proteins message (about 150 nts long) is required for efficient internal initiation of translation. Specifically, we have found that three stem-loops within the fist 50 nucleotides are essential for IRES-dependent translation initiation (Figure 1). Similar results were reported for the hsp70 IRES element of *Drosophila melanogaster* and *Homo sapiens*. In both cases, the complete sequence is required for cap-independent translation, since short deletions of the 5′UTR reduced IRES activity by 75% to 95% [35], [36].

The rich diversity of IRES structures in conjunction with their response to different conditions suggests the existence of multiple mechanisms driving IRES function [13], [17]. Therefore, it is very important to elucidate their RNA structure. Here we have used nucleotide modification by NMIA to analyze the secondary structure of the hsp101 IRES, a methodology that reports local flexibility of RNA. The maize hsp101 IRES secondary structure is the first experimental IRES structure reported for plant heat shock mRNAs (Figure 2). Structural domains identified in the maize hsp101 IRES include three stem-loops SL1 (9–21 nts), SL2 (27–34 nts) and SL3 (43–61 nts). This is followed by two large loops L1 (62–84 nts) and L2 (107–121 nts) flanked by 2 short stems, S1 and S2 (87–103 nts). Finally, a single-stranded region (126 to 150 nts) is located upstream of the AUG start codon (Figure 2). This RNA structure differs from other IRES elements [37]–[39]. In support of the biological relevance of this region, it is worth mentioning that the nucleotide sequence of this mRNA region is highly conserved among different *Zea mays* sequences deposited in databases, despite being outside of the coding region.

The relevance of RNA structure for IRES function is supported by the fact that deletions of the stem-loop SL1 in construct 101Δ17 abrogated translation. Likewise, the construct lacking the three stem-loops at the 5′end also failed to drive protein synthesis. This result suggests that a defective structure lacking SL1 is unable to interact with a *trans*-acting element. Conversely, the large single-stranded region presumably provides the binding site of proteins necessary to form the active translational complex under heat stress conditions, including HSP90 and the product of hsp101 message. Along this idea, recent data have shown that mRNAs associated with stress responses tend to have more single-stranded nucleotides at the 5′UTR, longer maximal loop length and higher free energy [40], as found in the RNA structure of the hsp101 IRES reported in the current study. On the other hand, it has been also noted that comparison of RNA structures of mRNA activated under stress response were poorly predicted *in silico*, emphasizing the need to determine the RNA structure biochemically.

Our results showed that the length and structure of the IRES is important for its function. However, IRES-binding proteins are also essential. Our experimental approach identified two proteins (50 kDa and 75 kDa) that were coprecipitated with the IRES element. The 75 kDa protein was identified as the molecular chaperone HSP90, a protein belonging to a family of ancient conserved factors found in all organisms including plants and animals [41], which has been reported to form stress granules and P-bodies under stress conditions [42]. This protein shows a large degree of amino acid conservation between animals and plants [43]. The active role of HSP90 to form the translational complex and initiate cap-independent translation was demonstrated by absence of translation of hsp101+1 in the presence of radicicol or the HSP90 antibody (Figure 4). HSP90 has also been reported to participate in the formation of the translational complex of the hepatitis C virus (HCV) IRES [44]; in this case, HSP90 interacts with the eIF3c subunit, preventing its ubiquitination and thus, impairing its degradation by the proteasome pathway.

Previous evidences has shown that eIF4G initiation factor is required for cap-independent translation driven by various IRES elements [13]. In the case of maize hsp101 IRES activity, eIFiso4G was more active than eIF4G. This result might be explained by the fact that the factor generally used for plant mRNA translation is eIFiso4G [30], [45] suggesting that this characteristic also prevails for IRES from plant mRNAs. This is in accordance with the fact that hsp101 IRES is specific to maize and thus, conserves a preference for factors from the same origin. Some cellular and viral IRES elements also require eIF4G factor [46]–[48] for translation initiation. However, there are no sequence similarities with the maize hsp101 IRES sequence, suggesting that the interaction can be based on a structural RNA domain, possibly helped by the interaction with other interacting factors.

Our results raised the possibility that SL1 could provide the binding-site for proteins such as HSP90 or eIFiso4G, a possibility that needs to be tested in the future. Interestingly, the results obtained in the current work suggest that transfer of the IRES hsp101 of maize to other plant mRNAs could confer translational advantage under heat stress and thus, alleviate problems of thermo-tolerance in plants.

In summary, this work reports the first experimental secondary structure of the IRES of a cellular mRNA of plants (hsp101), the participation of HSP90 in the maize IRES hsp101 complex and finally, the preference for eIFiso4G for translation initiation under cap-independent conditions.

## Material and Methods

### Constructs

The IRES sequence amplification reactions were performed as previously reported [2] using the PXE101 plasmid with the hsp101 cDNA as template including the 5′UTR region (Figure 1a). Recognition sequence for the restriction enzyme *SacI* was added to the primers used in the amplification reactions. Construct hsp101+1 was generated with the pair of primers forward hsp101+1 5′-CGAGCTCGCACAACATTTCAACCAGA-3′/reverse RV101* 5′-AGCAGCCATGAATCCGGACAACTCTCGACG-3′. For constructs hsp101Δ17 and hsp101Δ50, the pair of primers forward hsp101-A 5′-CGAGCTCGAAACACTAGCCGAAGCAA-3′/reverse RV101* and forward hsp 101-B 5′-CGAGCTCCACCTGGTGGGATCATCTC-3′/reverse RV101* respectively, were used. PCR products were inserted into the pGEM-T EASY cloning vector to generate pGEM-101+1, pGEM-101Δ17 and pGEM-101Δ50, respectively. The fragments were then excised with *SacI* to replace the IRES sequence in pBIC-5 generating pBIC-hsp101+1, pBIC-hsp101Δ17 and pBIC-hsp101Δ50 constructs. RNAs derived from pGEM-T constructs were synthesized using T7 RNA polymerase with Pst1 linearized plasmids. Bicistronic RNAs pBIC-5, 101+1, 101Δ17 and 101Δ50 synthesized *in vitro* using HpaI linearized plasmids and T7 RNA polymerase [2].

### *In vitro* translation

Cap-dependent and cap-independent translation was examined either in rabbit reticulocyte lysate (RRL) or Wheat Germ Extract (WGE). For cap-dependent translation analysis, a mix of RRL (17.5 µl), RNasin (1 µl), amino acids mix without methionine (1 µl), the bicistronic RNA (100 ng) and [^35^S] methionine (1 µl, 10 µCi) was prepared in a final volume of 35 µl with DEPC-treated water. The mix was incubated 60 min at 30°C; at the end of the reaction, 1µl of each sample was obtained and the synthesized proteins were resolved by SDS-PAGE. Gels were dried and exposed to a phosphorscreen to be analyzed by the QUANTITY ONE software (BIORAD). Values represent the mean (± SD) of three independent assays.

To generate the cap-independent RRL translation system, 50 ng of mRNA encoding the Lb protease of FMDV, a protein that cleaves eIF4G preventing cap-dependent translation [14], was added to the RRL mix and incubated at 30°C for 15 minutes. Subsequently, 100 ng of each bicistronic mRNA were added to the mix and incubated for another 45 minutes prior to analyze the translation products as indicated above. When indicated, the HSP90 inhibitors, Radicicol (SIGMA) and HSP90 antibody (Santa Cruz), or the commercially purified HSP90 protein (100 ng) (BIOMART) were added to the hsp101 IRES-driven translation in separate reactions.

Similarly, WGE was used either complete (cap-dependent) or eIF4E and eIFiso4E-depleted (cap-independent). For cap-independent translation, eIF4E and eIFiso4E were depleted from WGE using m^7^GTP sepharose [49]. To this end, 150 µl of m^7^GTP sepharose were centrifuged at 1500 rpm for 1 min to eliminate the supernatant and the resin was washed twice with 2 volumes of m^7^GTP buffer (100 mM KCl, 20 mM HEPES, pH7.6, 0.2 mM EDTA, 10% glycerol, 0.05 mM PMSF, 0.5 mM DTT). Then, 50 µl of WGE were added to the resin and the mix was incubated for 30 minutes at 4°C with gentle shaking. Finally, the sample was centrifuged at 1500 rpm for 1 min at 4°C, the supernatant was recovered and used as the WGE depleted lysate. The translation reaction mix contained 12.5 µl of WGE depleted, 1 µl RNasin, 1 µl of amino acids mix without methionine, 100 ng of hsp101+1 RNA, 1 µl [^35^S] methionine (10 µCi), 100 ng of eIF4G or eIFiso4G and DEPC-treated water to reach a volume of 25 µl. The mix was incubated for 60 min at 25°C. Newly synthesized proteins were resolved by SDS-PAGE loading 1 µl of each sample. Gels were dried and exposed to a phosphorimager screen.

### Determination of the maize hsp101 IRES secondary structure by Selective 2′-Hydroxyl Acylation Primer Extension (SHAPE)

In order to determine the IRES secondary structure, hsp101+1 RNA was synthesized with T7 RNA polymerase using pBIC-hsp101+1 plasmid linearized with *BbuI* [50]. SHAPE reactivity assays were carried out as described [27] with some modifications [28]. Briefly, hsp101+1 RNA (2 pmol) were resuspended in 12 µl 0.5X TE buffer, heated for 2 min at 95°C and then cooled for 2 min at 4°C; 6 µl of 3.3-RNA folding mix (333 mM HEPES, pH 8.0, 20 mM MgCl_2_, 333 mM NaCl) were added and incubated for 20 min at 37°C. This mix was divided into two tubes (9 µl in each tube); 1.5 µl NMIA (65 mM) [27] was added to one of the tubes (+) and 1.5 µl DMSO to the other (−). Either reaction was incubated for 45 min at 37°C. At the end of the incubation period, 90 µl H_2_O, 4 µl 5 M NaCl, 1 µl glycogen (20 mg/ml), 2 µl 100 mM EDTA, pH 8 and 350 µl absolute ethanol were added to each reaction tube. Samples were precipitated and the pelleted RNA was resuspended in 20 µl 0.5X TE. Ten microliters of each RNA sample, NMIA (+) and (−), were transferred to a 200 µl tube; 0.5 µl of the [^32^P]-5′ labeled oligonucleotide (GGCCTTTCTTTATGTTTTTGGCG) using polynucleotide kinase (PROMEGA) were added and incubated first, for 5 min at 65°C, then for 5 min at 35°C and finally at 4°C for 1 min. Three microliters of reverse transcriptase (RT) Superscript III buffer, 1U RNasin, 0.25 µl 100 mM DTT, 1.5 µl 10 mM dNTPs and 0.25 µl H_2_O were added to each sample and further incubated for 1 min at 52°C. Then, 0.25 µl Superscript III RT (INVITROGENE) was added and the mix and incubated for 30 min at 52°C. At the end of the reaction, 0.4 µl of 4 M NaOH were added to each sample. Samples were precipitated by adding 1 volume of AcNH4, 1 µl glycogen and 75 µl absolute ethanol. Pellets were resuspended in 10 µl H_2_O plus 4 µl formamide buffer (90% formamide). Primer extension products were resolved on denaturing 6% polyacrylamide 7 M urea gels, in parallel to a sequence ladder prepared with the same 5′end-labeled primer. Gels were dried and exposed to a radiography film for quantification. SHAPE reactivity data was analyzed as described [50].

### Purification of IRES-associated proteins

In order to identify the proteins interacting with hsp101 IRES (either sense hsp101+1 or antisense orientation inserted in the bicistronic constructs), 101+1, 101Δ17 and 101Δ50 RNAs (derived from pGEM-T EASY), were synthesized adding a poly-A tail using poly-A polymerase (AMBION) as described by the manufacturer. From each RNA sample, 2.5 µg were transferred to 100 µl of binding buffer (10 mM Tris, pH 7.5, 100 mM KCl, 2 mM MgCl_2_) containing 30 µl oligo dT sepharose. Samples were incubated for 30 min at 4°C with gentle shaking and then washed twice with binding buffer to remove unbound RNA. Then, 100 µg of RRL and 10 µg total RNA (nonspecific competitor) were added to a final volume of 100 µl binding buffer and incubated for 60 min at 4°C with gentle shaking. Samples were washed twice with binding buffer followed by washing buffer (10 mM Tris, pH 7.5, 100 mM KCl, 0.5 mM MgCl_2_) to remove unbound proteins to the IRES-oligo dT sepharose complex. IRES-protein complexes were resolved by SDS-PAGE and visualized by silver staining in triplicate experiments. Thin slices containing differential IRES-bound proteins were excised and identified by mass spectrometry (LC/MS/MS) at Quebec Genomics Center.
